# Deciphering the Origin and Evolution of the X_1_X_2_Y System in Two Closely-Related *Oplegnathus* Species (Oplegnathidae and Centrarchiformes)

**DOI:** 10.3390/ijms20143571

**Published:** 2019-07-22

**Authors:** Dongdong Xu, Alexandr Sember, Qihui Zhu, Ezequiel Aguiar de Oliveira, Thomas Liehr, Ahmed B. H. Al-Rikabi, Zhizhong Xiao, Hongbin Song, Marcelo de Bello Cioffi

**Affiliations:** 1 Key Lab of Mariculture and Enhancement of Zhejiang Province, Marine Fishery Institute of Zhejiang Province, Zhoushan 316100, China; 2 College of Fisheries, Zhejiang Ocean University, Zhoushan 316100, China; 3 Laboratory of Fish Genetics, Institute of Animal Physiology and Genetics, Czech Academy of Sciences, Rumburská 89, 277 21 Liběchov, Czech Republic; 4 Departamento de Genética e Evolução, Universidade Federal de São Carlos (UFSCar), Rodovia Washington Luiz Km. 235, C.P. 676, São Carlos SP 13565-905, Brazil; 5 Secretaria de Estado de Educação de Mato Grosso—SEDUC-MT, Cuiabá MT 78049-909, Brazil; 6 University Clinic Jena, Institute of Human Genetics, 07747 Jena, Germany; 7 Laboratory for Marine Biology and Biotechnology, Institute of Oceanology, Chinese Academy of Sciences, 7 Nanhai Road, Qingdao 266071, China

**Keywords:** comparative genomic hybridization, centric fusion, multiple sex chromosomes, *Oplegnathus*, whole chromosome painting

## Abstract

*Oplegnathus fasciatus* and *O*. *punctatus* (Teleostei: Centrarchiformes: Oplegnathidae), are commercially important rocky reef fishes, endemic to East Asia. Both species present an X_1_X_2_Y sex chromosome system. Here, we investigated the evolutionary forces behind the origin and differentiation of these sex chromosomes, with the aim to elucidate whether they had a single or convergent origin. To achieve this, conventional and molecular cytogenetic protocols, involving the mapping of repetitive DNA markers, comparative genomic hybridization (CGH), and whole chromosome painting (WCP) were applied. Both species presented similar 2n, karyotype structure and hybridization patterns of repetitive DNA classes. 5S rDNA loci, besides being placed on the autosomal pair 22, resided in the terminal region of the long arms of both X_1_ chromosomes in females, and on the X_1_ and Y chromosomes in males. Furthermore, WCP experiments with a probe derived from the Y chromosome of *O*. *fasciatus* (OFAS-Y) entirely painted the X_1_ and X_2_ chromosomes in females and the X_1_, X_2_, and Y chromosomes in males of both species. CGH failed to reveal any sign of sequence differentiation on the Y chromosome in both species, thereby suggesting the shared early stage of neo-Y chromosome differentiation. Altogether, the present findings confirmed the origin of the X_1_X_2_Y sex chromosomes via Y-autosome centric fusion and strongly suggested their common origin.

## 1. Introduction

The marine fish family Oplegnathidae (order Centrarchiformes) includes only one genus *Oplegnathus*, which is currently composed of seven extant species [1]. Two of them, *O*. *fasciatus* and *O*. *punctatus*, are commercially valuable taxa in East Asia, representing important fishery resources for offshore cage aquaculture [2]. Extensive efforts have been therefore undertaken to exploit their genetics and genomics, including insights into their populational genetic structure, with an aim to foster the technological advancements in their aquaculture [3,4,5,6]. One of the marked features of both species’ genomes is the presence of a multiple ♀X_1_X_1_X_2_X_2_/♂X_1_X_2_Y sex chromosome system [7,8], which might have potentially some bearing to the observed sexual dimorphism in growth and/or possibly to other traits that are important for the fish breeding industry.

Male karyotypes of *O*. *fasciatus* and *O*. *punctatus* are composed of 2n = 47 chromosomes (1m + 2m/sm + 44a), while females possess 2n = 48 chromosomes (2m/sm + 46a) [7,8,9]. The mapping of distinct microsatellite DNA motifs through fluorescence *in situ* hybridization (FISH) uncovered a specific accumulation of some of them on the large metacentric Y chromosome of *O*. *fasciatus* [10], while Li et al. [9] reported no association between the location of 5S and 18S rDNA and sex chromosomes of *O*. *punctatus*. Although these reports provided a preliminary description of the multiple sex chromosome system in *Oplegnathus*, more detailed studies are needed to clarify its origin and molecular composition. 

Neo-sex chromosome systems usually arise from structural rearrangements (typically fusions or reciprocal translocations) between autosomes and original sex chromosomes, or through the fission/fragmentation or nondisjunctions involving solely the original sex chromosome pair [11,12,13,14,15,16,17,18]. Except for situations when autosomal segments are equally added to both chromosomes from the original sex pair, or when neo-sex chromosomes emerge via transition between the XY and ZW sex chromosome systems (forming in both cases either neo-XY or neo-ZW, e.g., [19,20,21,22]), the acquisition of neo-sex chromosomes concomitantly means the emergence of a multiple sex chromosome system, which is usually cytogenetically well recognizable, as it creates different numbers of chromosomes between sexes [23]. The investigation of sex chromosome differentiation and evolution is a very attractive research area of contemporary genetics and evolutionary biology, and teleost fishes represent one of the most vital model groups for its investigation, largely due to the relative evolutionary “youth” of their sex chromosomes, allowing early stages of their differentiation to be analyzed [24,25,26,27,28,29]. 

Including *Oplegnathus*, about 60 cases of multiple sex chromosomes have so far been reported across the teleost phylogeny (reviewed in [13], for more recent examples, see [30,31,32,33,34,35,36,37,38,39]). In this context, molecular cytogenetics provides a powerful toolbox for understanding the genome evolution and organization [40,41,42], and many of these approaches have enabled unique insights into the vertebrate sex chromosome evolution. More specifically, the genomic *in situ* hybridization (GISH) and comparative genomic hybridization (CGH) (methods employing whole-genomic DNA probes to compete for hybridization on an investigated chromosome complement) have repeatedly proven to be efficient in identifying homomorphic sex chromosomes and have permitted a more thorough delimitation of region of differentiation on the heteromorphic sex chromosomes by uncovering sex-specific repetitive DNA accumulation [16,43,44,45,46,47,48,49,50,51]. Besides CGH, whole chromosome painting (WCP), which employs the chromosome-specific probes, has contributed to the knowledge of the evolution of supernumerary and sex chromosomes by identifying several chromosomal rearrangements, including those leading to the emergence of multiple sex chromosomes [12,17,31,42,49,52,53,54,55,56,57]. 

In the present study, we aimed to scrutinize the evolutionary processes linked to the establishment of a multiple X_1_X_2_Y sex chromosome system in two closely-related fish species as well as to delimit the stage and extent of its differentiation and whether this sex chromosome system originated from the same linkage groups in both cases. To achieve this, we performed an extensive cytogenetic investigation in *O*. *fasciatus* and *O*. *punctatus*, using conventional cytogenetic protocols (Giemsa-staining and C-banding), the mapping of repetitive DNAs, comparative genomic hybridization (CGH), and whole chromosome painting (WCP).

## 2. Results

### 2.1. Karyotype Analysis and Distribution of Constitutive Heterochromatin

**Figure 1:**
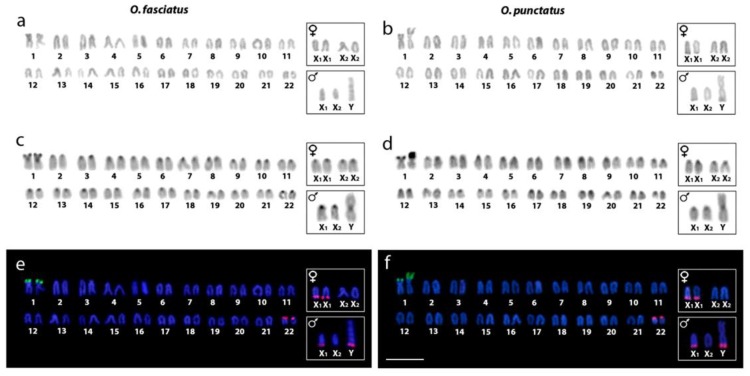
Karyotypes of males and females of *O*. *fasciatus* (**a**,**c**,**e**) and *O*. *punctatus* (**b**,**d**,**f**) after different cytogenetic protocols. Giemsa staining (**a**,**b**); C-banding (**c**,**d**), and dual-color fluorescence hybridization (FISH) with 18S (green) and 5S (red) rDNA probes (**e**,**f**). Chromosomes were counterstained with 4′,6-diamidino-2-phenolindole (DAPI; blue). Insets depict male and female sex chromosomes. Scale bar = 5 μm.

The karyotypes of both species were composed of 2n = 47 chromosomes in males (1m + 2m/sm + 44a) and 2n = 48 in females (2m/sm + 46a), indicating the presence of a X_1_X_2_Y multiple sex chromosome system (Figure 1a,b). These data are in accordance with previous reports [7,8,9], with few deviations related to chromosomal morphology and the numbering of chromosome pairs (see Discussion). The male-specific Y chromosome corresponded to the largest metacentric element in the karyotype, hence being easily recognizable already after Giemsa staining. Both the X_1_ and X_2_ chromosomes were acrocentrics of a similar size and their precise identification in the conventionally stained karyotype is therefore difficult to assess with any degree of confidence. Therefore, sex chromosomes were placed in a separate box (Figure 1a,b). C-banding revealed a predominant location of constitutive heterochromatin in the centromeric regions of all chromosomes, with conspicuous blocks being located on the short arms of pair No. 1 in both species (Figure 1c,d), where they coincide with nucleolar organizer regions (NORs). A remarkable size heteromorphism of this single NOR site was observed in males and females of *O*. *punctatus*, but not in *O*. *fasciatus* (Figure 1e,f; Supplement, Figure S1). 

### 2.2. Chromosomal Mapping of Repetitive DNA Markers

The distribution of 5S and 18S rDNA sites was identical in the haploid complement of both sexes, except for the occurrence of additional 5S rDNA cistron on the Y chromosome in males. While the 18S rDNA probe marked a single site with a very intense signal located in the short arms of chromosome pair No. 1 in both species, the 5S rDNA probe consistently revealed four clusters in both sexes, but with differences in their location. While two 5S rDNA loci occupied the short arms of the smallest pair, No. 22 in both sexes, the two remaining ones were found in the terminal regions of q arms of both X_1_ chromosomes in females, and on a single X_1_ and Y chromosome in males (Figure 1e,f). Nonetheless, as it cannot be unambiguously decided from the available data whether 5S rDNA resides in the original X chromosome (X_1_) or in the newly involved autosomal homolog (X_2_), the placement of this 5S rDNA loci is only tentative and other data will be necessary to fully address this question. 5S rDNA patterns reported here deviate significantly from the one reported by Li and colleagues [9] in *O*. *punctatus*, which will be later discussed in detail.

**Figure 2:**
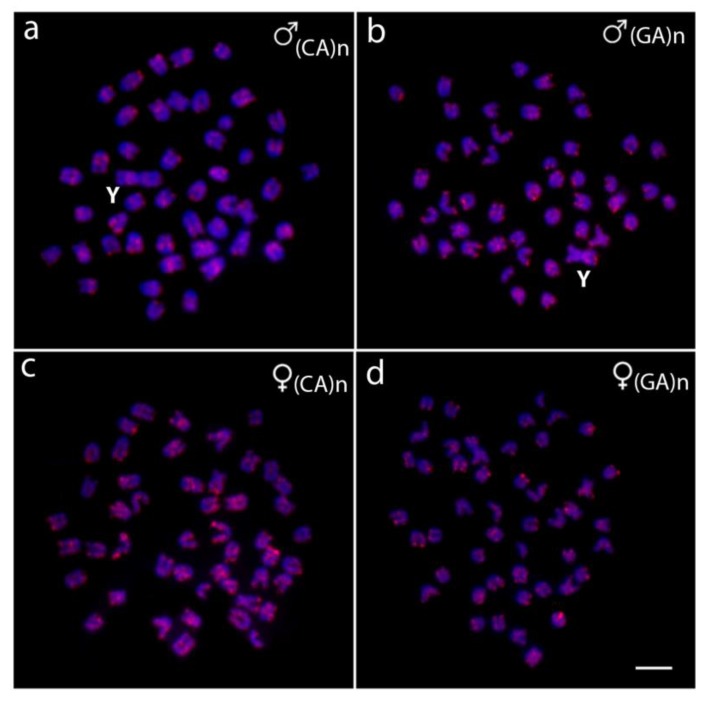
Mitotic chromosome spreads of *O*. *punctatus* males (**a**,**b**) and females (**c**,**d**) hybridized with the microsatellite probes (CA)_15_ (**a**,**c**) and (GA)_15_ (**b**,**d**). Chromosomes were counterstained with DAPI (blue). Scale bar = 5 μm.

The chromosomal mapping of the microsatellite motifs (CA)_15_ and (GA)_15_ performed in *O*. *punctatus* showed a scattered distribution for both repeats throughout the whole chromosome complement, although distinct accumulations were apparent, showing a strong preference for the telomeric regions. No unique accumulations were observed on the sex chromosomes (Figure 2). Regarding *O*. *fasciatus*, the hybridization patterns of (CA)_15_, (GA)_15_, and several other microsatellite motifs have been previously described [58].

**Figure 3:**
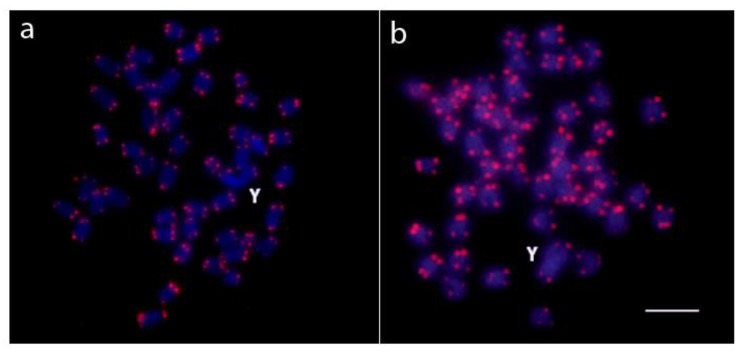
Metaphase plates of *O*. *fasciatus* (**a**) and *O*. *punctatus* (**b**) showing the location of telomeric (TTAGGG)_n_ repeats. Chromosomes were counterstained with DAPI (blue). Bar = 5 µm.

FISH with the canonical vertebrate telomere repeat (TTAGGG)_n_ revealed, as expected, positive hybridization to the telomeres of all chromosomes in both species and no additional interstitial telomeric sites (ITSs) were detected (Figure 3).

### 2.3. Characterization of Male vs. Female Genome Differences by CGH

**Figure 4:**
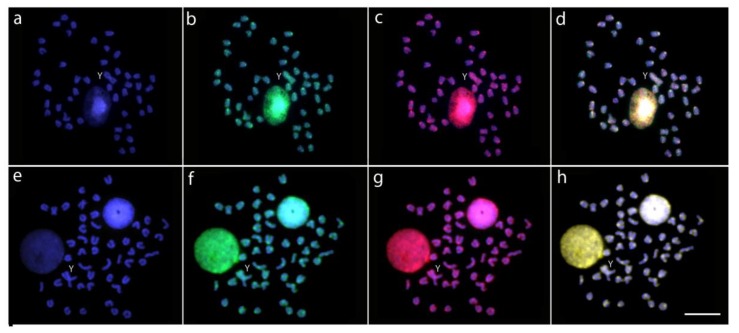
Mitotic chromosome spreads of males of *O*. *fasciatus* (**a**–**d**) and *O*. *punctatus* (**e**–**h**) after male vs. female comparative genomic hybridization (CGH) experiments. The first column (**a**,**e**): DAPI images (blue); second column (**b**,**f**): Hybridization pattern of the female-derived probe (green) of each analyzed species; third column (**c**,**g**): Hybridization pattern of the male-derived probe (red) of the respective species. The fourth column (**d**,**h**): Merged images of both genomic probes and DAPI staining. The common genomic regions for male and female are depicted in yellow. The Y chromosome is indicated. Scale bar = 10 µm

The intraspecific genomic hybridization between males and females against the background of the male chromosome complement revealed no exclusive accumulations of male-specific or male-enriched repetitive sequences either on the neo-Y chromosome or in the rest of the karyotype in both species (Figure 4). At the same time, in both species the genome-derived probes showed preferential localization in centromeric and pericentromeric regions of most/all chromosomes and in the terminal parts of some elements, where they equally hybridized (yellow signals, i.e., combination of green and red), matching the C-banding pattern and thus indicating repetitive content of these regions.

### 2.4. Detection of Chromosomal Homologies by WCP Experiments

**Figure 5:**
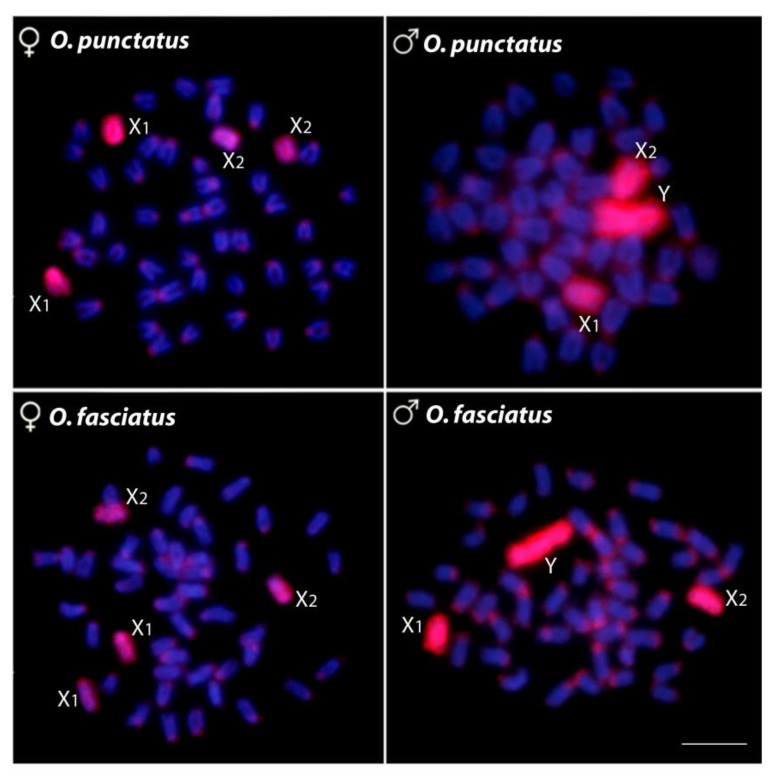
Chromosome painting with the OFAS-Y probe (red) applied onto male and female metaphase chromosomes of *O*. *fasciatus* and *O*. *punctatus*. Chromosomes were counterstained with DAPI (blue). Note that the OFAS-Y probe completely painted the Y-chromosomes, as well as the X_1_ and X_2_ chromosomes in both species. Scale bar = 5 μm.

The WCP experiments with the OFAS-Y painting probe completely stained the X_1_ and X_2_ chromosomes in females and the X_1_, X_2_, and Y-chromosomes in the males of both species, thus confirming the orthology of both X_1_X_2_Y sex chromosome systems (Figure 5). 

## 3. Discussion

Both *Oplegnathus* species studied herein are evolutionarily closely related and share the same 2n and karyotype structure. Our analysis agreed with previous reports [7,8,58], with only slight deviations with respect to our previous study [58] where we identified a single submetacentric pair (No. 1) as a metacentric one. Such an incongruence may reflect either the placement of this chromosome pair on the borderline between both chromosome categories or it may have resulted from the description of slightly karyotypically different populations. The latter explanation may be also applied on the subtle differences between C-banding patterns reported here and in the study of Li et al. [9]

A marked feature of karyotypes of both *Oplegnathus* species is the presence of an X_1_X_2_Y sex chromosome system, which may be inferred from a difference in 2n between males (2n = 47) and females (2n = 48). Such a scenario strongly favors a centric fusion as an underlying mechanism ([58], present study). More specifically, a centric fusion involving one homolog from each of the two non-homologous acrocentric pairs gave rise to a large neo-Y chromosome, with the remaining unpaired homologs corresponding to the neo-X_1_ and neo-X_2_ chromosomes in the male karyotype (Figure 1). In both species under study, the location of 5S rDNA sequences at the telomeric position of the X_1_ and Y-chromosomes in males and in both X_1_ chromosomes in females, serves as a relevant marker supporting such a scenario. Interestingly, the previous study of Li et al. [9], revealed only a single 5S rDNA-bearing chromosome pair (the smallest pair No. 22 in the present study) in the chromosome complement of *O*. *punctatus*. It may be that these authors studied a different population of *O*. *punctatus*, with an altered 5S rDNA pattern. Alternatively, Li et al. [9] employed a somewhat different rDNA probe composition and/or different conditions for hybridization and stringent washing, which might have potentially eliminated the hybridization to loci that had already accumulated a certain degree of sequence divergence (which may count, for instance, for the loci that correspond to the pseudogenic variants). 

The association between rDNAs and the sex chromosomes has been increasingly evidenced in fishes during the last decade, including taxa with multiple sex chromosomes [31,33,38,49,56,59,60,61,62,63,64]. As analogous examples have repeatedly been documented in other animals, different authors speculated about diverse potential roles for rDNA on standard or neo-sex chromosomes, including the effects on the recombination frequency (which may be lowered in nearby chromosomal regions [45,65,66]), prevention against the complete loss of the degenerating sex chromosome due to the persistent presence of structural genes [66], proper pairing and segregation of sex chromosomes [22,67] or as a boundary that prevents the spreading of inactivation on neo-sex chromosome from the original segment to a newly added pseudo-autosomal material [55]. Given the position of rDNA clusters on *Oplegnathus* sex chromosomes, we may entirely exclude the last mentioned possibility. 

It is also noteworthy that within the range of standard fish sex chromosome systems, 5S rDNA was found to be scattered exclusively along the entire length of the W chromosome in *Aulopus japonicus* [68] or to reside exclusively within the sex-specific region on the W chromosome of *Triportheus signatus* [47], as well as on the Y_1_ chromosome of *Hoplias malabaricus*, karyomorph G [49]. Association of rDNA with sex-determining region seems also improbable in *Oplegnathus* as (i) the previous report of Li et al. [9] do not show any sex-linked 5S rDNA loci in their sampling despite the presence of a X_1_X_2_Y sex chromosome system, (ii) neither CGH results support such a scenario (see below), and (iii) the establishment of the sex-determining region may have occurred rather around the fusion point on neo-Y [69] instead of the telomeric regions. It is rather likely that rDNA clusters have no bearing to sex chromosome differentiation in this case and that they follow their own evolutionary dynamics [70]. 

Another cytogenetic marker valuable for tracking the evolutionary forces behind the creation of neo-sex chromosomes and especially for those with fusions as an underlying mechanism, is the mapping of telomeric sequences [71]. Their presence inside the chromosomes (as ITSs), in addition to their natural locations at chromosome ends, may serve as a hallmark of previous structural rearrangements [72]. ITSs have been identified in differentiated sex chromosomes of several animal species, highlighting the chromosomal rearrangements related to their origin [61,71,73,74] and they have also been clearly evidenced in multiple sex chromosomes of several fish taxa (e.g., [61,62,63,75,76]), while they were lacking in others despite the products of certain rearrangements were obvious [30,53,77]. The latter scenario fits well to both *Oplegnathus* species under study. It is well known that the process of chromosome fusion might follow mechanistically several scenarios based on the location of DNA breakpoints. It seems that in *Oplegnathus*, telomere sequences have been either entirely eliminated during the process of fusion, or they have been retained but the residual traces of ITSs have already been lost from the fusion points or have been reduced to a very low copy number undetectable by FISH analyses [71,78,79] (Figure 3). The C-banding data support this inference because the heterochromatic segment observed on the X chromosome was not significantly extended on the neo-Y chromosome (Figure 1). 

Microsatellites are highly dynamic repetitive sequences, therefore they are useful for analyzing the evolutionary dynamics linked to karyotype diversification on a sub-chromosomal level [40,80,81] as well as the sex chromosome differentiation [82,83,84,85]. Although both dinucleotide motifs (GA)_15_ and (CA)_15_ were present on the Y chromosomes, no exclusive or biased accumulations were observed on the sex chromosomes in comparison with the autosomes of both species (present study, see Figure 2; and [58]). These findings agree with the general patterns found in the majority of fish multiple sex chromosomes, where little or no differential accumulation of heterochromatin and repetitive DNA sequences accompanies their emergence and differentiation [25,32,33,37,38,40,45] and it also corroborates our findings yielded by the CGH method (see below).

CGH experiments have been used to uncover the sex-specific regions among the gonosomes of many animal species [48,49,86,87,88]. The reproducibility of the CGH method largely relies on the presence of genome-specific or genome-enriched accumulations of repetitive DNA [89,90]. In the case of male vs. female comparisons, it is expected to reveal the specific repetitive DNA accumulations in the heterogametic sex, i.e., either on the Y or W chromosome, which may provide a clue about a degree of differentiation inside the sex-specific region. Here, CGH procedures were not sensitive enough to reveal any putative sex-specific region on the neo-Y chromosomes in either *Oplegnathus* species. This observation has at least two possible explanations. First, the emerging male-specific region did not have yet enough time to undergo significant degeneration through repetitive DNA accumulation and sequence divergence, which may suggest a relatively recent origin of the X_1_X_2_Y system in both species. However, it has also been reported that very young neo-sex chromosomes with extensive repetitive DNA accumulations may appear among diverse organisms (e.g., [91,92,93]). Second, the male-specific region in both *Oplegnathus* species may be of a small size (regardless its age) and may thus remain below the resolution limit of the CGH method. Given that the CGH may detect regions of divergence not smaller than approximately 2–3 Mb (megabases) [94] and that many well-characterized fish sex-determining regions encompass only one or just a few Mb and sometimes hardly a few Kb (kilobases) (e.g., [28,95,96,97,98]) or even less [99], it is highly likely that such region would escape our attention in *Oplegnathus*. It seems that fish neo-sex chromosomes may accumulate small alterations within a small area of suppressed recombination and yet their emergence might have a significant impact on species divergence [100,101]. Among fishes, CGH have so far uncovered regions of marked differentiation only on neo-sex chromosomes of *Hoplias malabaricus* of karyomorph G [45,49] and in *Pyrrhulina semifasciata* [102], while it failed to show similar regions in males of *H*. *malabaricus* of karyomorph D [45]. It has been hypothesized that low differentiation accompanied with a very limited accumulation of repetitive DNA and heterochromatin on fish neo-sex chromosomes may be directly linked with a proper and stable trivalent formation and its subsequent segregation during the first meiotic division [75,77,103]. In this context, it would be desirable to investigate in further studies not only the sequence differentiation but also the epigenetic landscape of fish neo-sex chromosomes, as these patterns may differ significantly, with a notable example reported in grasshoppers [22]. 

WCP experiments using Y-specific probes applied to *O*. *fasciatus* and *O*. *punctatus* chromosomes confirmed the origin of multiple X_1_X_2_Y sex systems through a centric fusion of ancestral Y chromosome with an autosome, creating the large neo-Y chromosome. The results also gave strong evidence for its shared origin from the same linkage groups, which consequently makes it highly probable that these sex chromosomes evolved from the same evolutionary event. Multiple sex chromosomes may arise and get fixed either by the action of genetic drift in small isolated populations [49,57,104] or through the effects of a selection on particular traits (sexually-antagonistic alleles or the newly established linkage of certain genes with significant impact on local adaptation or speciation) (e.g., [21,69,100,105,106,107]). Sharing the same sex chromosome system involving orthologous chromosomes in closely-related species is particularly uncommon in fishes, where high frequency of sex chromosome lability and turnovers even among closely-related species or within species/species complexes has been abundantly reported [25,29,108,109,110,111,112], including WCP-based reports on closely-related fish taxa with neo-sex chromosomes [31,49,53,110,113,114]. In the *Oplegnathus* species studied herein, it seems that the shared X_1_X_2_Y sex chromosome system might have originated in the common ancestor of both species, similarly to what has been inferred for threespine stickleback *Gasterosteus aculeatus* [100,105]. To develop a more informed and comprehensive picture on this issue, additional finer-scale genome-wide studies are needed. 

## 4. Conclusions

In conclusion, the present data bring novel insights into the karyotype and sex chromosome differentiation in *O*. *fasciatus* and *O*. *punctatus*, which allowed us to track the underlying evolutionary processes and to shed light on the origin and differentiation of a multiple X_1_X_2_Y sex chromosome system. The genus *Oplegnathus* proved to be a vital fish taxon which may provide a useful opportunity to study the evolution of sex chromosomes and sex determination. Further investigations aiming at in-depth sequence and epigenetic analysis will advance our understanding of sex determination in these species.

## 5. Materials and Methods

### 5.1. Animals

A total of 8 males and 7 females of *O*. *fasciatus* and 6 males and 8 females of *O*. *punctatus* were collected from the research station of Marine Fishery Institute of Zhejiang Province (Xishan Island, City of Zhoushan, China). The experiments were approved by the Animal Ethics Committee of Zhejiang Ocean University and Marine Fishery Institute of Zhejiang Province (Process Number 2017C04003)

### 5.2. Chromosome Preparation and Analysis of Constitutive Heterochromatin

Mitotic chromosomes were obtained from kidney cells following the protocol described in Bertollo et al. [115]. For conventional cytogenetic analysis, chromosomes were stained with 5% Giemsa solution (pH 6.8). The distribution of constitutive heterochromatin was detected by C-banding according to Sumner [116]. All the experiments followed ethical protocols and anesthesia with clove oil was used, prior to sacrificing the animals so as to minimize suffering. The process was approved by the Animal Ethics Committee of Zhejiang Ocean University and Marine Fishery Institute of Zhejiang Province based on the Ethics of Animal Experimentation of the National Research Council. 

### 5.3. FISH with Repetitive DNA Sequences

The 5S rDNA probe included 120 base pairs (bp) of the 5S rDNA gene-coding region and the 200 bp long non-transcribed spacer (NTS) [117]. The 18S rDNA probe encompassed a 1400 bp long segment of the 18S rDNA coding region [118]. The 18S and 5S rDNA probes were labeled with Aminoallyl-dUTP-Atto-488 and Aminoallyl-dUTP-Atto-550, respectively, using the Nick-translation labeling kit (Jena Bioscience, Jena, Germany) according to the manufacturer’s instructions. Two microsatellite motifs with sequences (CA)_15_ and (GA)_15_ were directly labeled with Cy3 during the synthesis according to Kubát et al. [119]. FISH, for all mentioned repetitive sequences, was performed under high stringency conditions as described in Yano et al. [47]. Telomeric (TTAGGG)_n_ sequences were mapped using the Telomere PNA FISH Kit/Cy3 (DAKO, Glostrup, Denmark).

### 5.4. Preparation of Probes for Comparative Genomic Hybridization (CGH)

As the aim of this approach was to decipher a molecular composition and potential sex-specific accumulation of repetitive DNA on the X_1_X_2_Y sex chromosomes found in both species under study, the experimental scheme involved male vs. female intraspecific comparisons. For this, male and female genomic DNAs (gDNAs) of *O*. *fasciatus* and *O*. *punctatus* were isolated by the standard phenol-chloroform-isoamyl alcohol method [79]. While male gDNAs were labeled with Aminoallyl-dUTP-Atto-550, female gDNAs were labeled with Aminoallyl-dUTP-Atto-488. The labeling was performed by the Nick-translation labeling kit (Jena Bioscience, Jena, Germany). The final hybridization mixture for each slide contained 500 ng of both male- and female-derived labeled gDNA and 25 μg of unlabeled female-derived C_0_t-1 DNA of each respective species (to block the abundant repetitive sequences; prepared according to Zwick et al. [120]), dissolved in 20 μL of the hybridization buffer (50% formamide, 2× SSC, 10% SDS, 10% dextran sulfate and Denhardt’s buffer, pH 7.0). CGH experiments were carried out according to Symonová et al. [121]. 

### 5.5. Chromosome Microdissection, Probe Preparation, and Labeling

A total of 12 copies of the Y chromosome of *O*. *fasciatus* (hereafter designated as OFAS-Y) were manually microdissected using glass needles, under an inverted microscope (Zeiss Axiovert 135). The chromosomes were amplified by degenerate oligonucleotide primed-PCR (DOP-PCR), following the protocol described in Yang et al. [122]. Next, 1 μL of the primary amplification product was used as a template DNA in the secondary labeling DOP-PCR with Spectrum Orange-dUTP (Vysis, Downers Grove, USA) in 30 cycles, following Yang et al. [122]. The final probe mixture for one slide contained 500 ng of the OFAS-Y probe co-precipitated with 30 µg of C_0_t-1 DNA isolated from the *O*. *fasciatus* female genome and 30 µg of C_0_t-1 DNA isolated from the *O*. *punctatus* female genome. The hybridization procedures were done following Yano et al. [47]. 

### 5.6. Microscopy and Image Processing

At least 30 metaphase spreads per individual were analyzed to confirm the diploid number (2n), karyotype structure, and FISH results. Images were captured using an Olympus BX50 microscope (Olympus Corporation, Ishikawa, Japan) with CoolSNAP and the images were processed using Image Pro Plus 4.1 software (Media Cybernetics, Silver Spring, MD, USA). Final images were optimized and arranged using Adobe Photoshop, version 7.0. 

## Supplementary Materials

Supplementary materials can be found at https://www.mdpi.com/1422-0067/20/14/3571/s1.
