# Supplementary figures and images for: Deciphering the Origin and Evolution of the X1X2Y System in Two Closely-Related Oplegnathus Species (Oplegnathidae and Centrarchiformes)
Source: Int J Mol Sci. 2019 Jul 22;20(14):3571. doi: 10.3390/ijms20143571 (PMC6678977; doi:10.3390/ijms20143571)

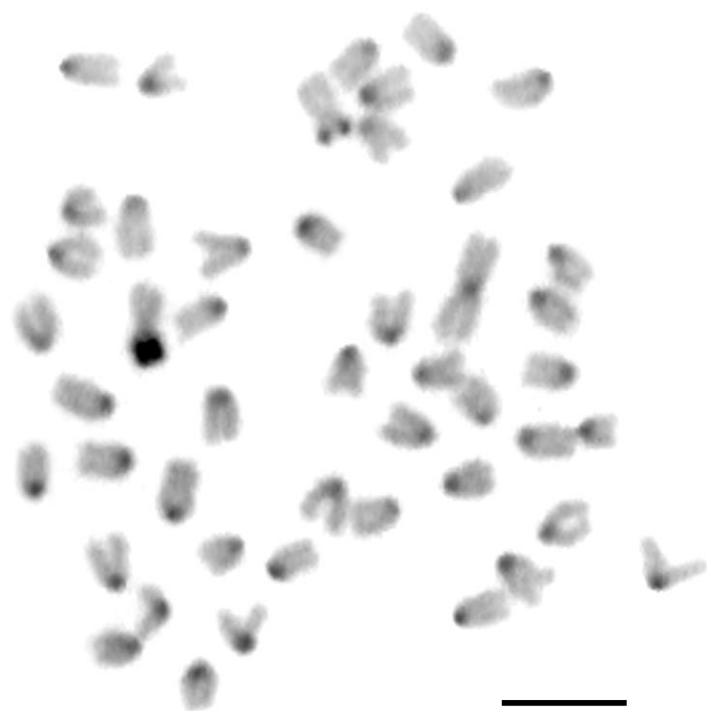

Supplement: Supplementary file 1 [file ijms-20-03571-s001.pdf]
